# Leptin promotes melanoma tumor growth in mice related to increasing circulating endothelial progenitor cells numbers and plasma NO production

**DOI:** 10.1186/1756-9966-30-21

**Published:** 2011-02-21

**Authors:** Fatemehsadat Amjadi, Shaghaygh Haghjooy Javanmard, Hamid Zarkesh-Esfahani, Majid Khazaei, Manijeh Narimani

**Affiliations:** 1 Applied PhysiologyResearchCenter and Department of Physiology, School of Medicine, Isfahan University of Medical Sciences, Isfahan, Iran; 2 Department of Immunology, School of Medicine, Isfahan University of Medical Sciences, Isfahan, Iran; 3 Department of physiology, School of Medicine, Isfahan University of Medical Sciences, Isfahan, Iran

## Abstract

**Background:**

Epidemiological studies propose that obesity increases the risk of several cancers, including melanoma. Obesity increases the expression of leptin, a multifunctional peptide produced predominantly by adipocytes which may promote tumor growth. Several recently experiments have suggested that the tumors growth is in need of endothelial progenitor cell (EPC) dependent generation of new blood vessels.

Our objectives in the present study were to examine the effects of leptin on melanoma growth, circulating EPCs number and plasma levels of nitric oxide metabolites (NOx).

**Methods:**

2 × 10^6 ^B16F10 melanoma cells were injected to thirty two C57BL6 mice subcutaneously. The mice were randomly divided into 4 groups (n = 8) in 8th day. Two groups were received twice daily intraperitoneal(i.p) injections of either PBS or recombinant murine leptin (1 μg/g initial body weight). Two groups were received i.p. injections of either 9F8 an anti leptin receptor antibody or the control mouse IgG at 50 μg/mouse every 3 consecutive days. By the end of the second week the animals were euthanized and blood samples and tumors were analyzed.

**Results:**

The tumor weight, EPC numbers and NOx level in leptin, PBS, 9F8, and IgG group were (3.2 ± 0.6, 1.7 ± 0.3, 1.61 ± 0.2,1.7 ± 0.3 g), (222.66 ± 36.5, 133.33 ± 171, 23.33 ± 18, 132.66 ± 27.26/ml of blood), and (22.47 ± 5.5, 12.30 ± 1.5, 6.26 ± 0.84, 15.75 ± 6.3 μmol/L) respectively. Tumors weight and size, circulating EPC numbers and plasma levels of NOx were significantly more in the leptin than 9f8 and both control groups (p < 0.05). The plasma concentration of NOx significantly decreased in 9f8 treated mice compare to control group (p < 0.05).

**Conclusions:**

In conclusion, our observations indicate that leptin causes melanoma growth likely through increased NO production and circulating EPC numbers and consequently vasculogenesis.

## Introduction

Tumor growth and metastasis is dependent on the formation and assembly of new blood vessels [1]. Several recent experiments have suggested that the growth of some types of tumors is not only dependent on angiogenesis (i.e., mature endothelial-cell dependent generation of new blood vessels) but also is associated with vasculogenesis, which means endothelial progenitor cell (EPC) dependent generation of new blood vessels [2].

Mobilization of EPCs from the bone marrow constitutes a critical step in the formation of de novo blood vessels, and levels of peripheral blood EPCs have been shown to be increased in certain malignant states.

Furthermore, inhibition of EPCrecruitment in neoplastic conditions has been efficiently attenuated tumors growth and progression [3-6]. In this regard, EPCs holds potential pathophysiological role in melanoma and may offer a potentialpredictive indicator of tumor growth and progression.

Leptin, a product of the obese (*ob*) gene, is a multifunctional peptide produced predominantly by adipocytes[7]. Besides itsseveral pleiotropic effects including regulation of food intake and energy expenditure, reproductionand immunefunctions, leptin has been found to exerts angiogenic effects in vitro and in vivo, which are mediated by enhancement of the endothelium derived nitric oxide (NO) production[8,9], the expression of vascular endothelial growth factor (VEGF) and VEGF-receptor 2 and activation of endogenous fibroblasticgrowth factor -2 [10,11].

The leptin receptor (ObR) is expressed on various cell types, including endothelial cells,[12,13] CD34-positive hematopoietic cells,[14] and peripheral blood-derived early and lateoutgrowth endothelial progenitor cells [15,16]. Furthermore leptin increased the adhesion, transmigration, and incorporation of early outgrowth progenitor cells into experimental arterial lesions [15].

Nitric oxide (NO) is recognized as an important final target of leptin effecton the endothelium. Leptin can induce NO formation by directly activating endothelial NO synthase through the Akt pathway[17,18].

Leptin receptors are expressed in mouse melanoma cells, but there is very little previous information on the relationship between leptin and melanoma. One epidemiological study reported that high serum leptin was positively correlated with melanoma risk [19]. Moreover, it has been shown that leptin directly accelerated melanoma tumor growth in mice [20].

In the present study, we hypothesized that the leptin may increase the EPC numbers and NO production in peripheral blood of melanoma tumor bearing mice.

## Methods

### Cell culture

B16-F10 melanoma cells which can grow in the C57BL/6 strain mouse were purchased from the National Cell bank of Iran (NCBI, Pasteur institute of Iran). Cells were cultured in DMEM supplemented with 4 mM L-glutamine, 4.5 g/l glucose, 10% FBS, and antibiotics (100 μg/ml streptomycin, 100 μg/ml penicillin) under humidified air with 5% CO2 at 37°C.

After 80% confluency of the melanoma cell monolayer in culture, the cells were washed and detached with PBS containing 0.25% trypsin and 0.03% EDTA and then pelleted by brief centrifugation at 100 *g*. The supernatant was removed, cell pellets were resuspended in PBS, and the cell number was counted.

### Animal experiments

Six to 8 week-old male C57BL/6 mice were purchased from Pasteur institute of Iran and served as recipient mice for tumor inoculation. Mice were permitted 1 week to acclimate to the environment before experiment. All mice were treated according to the guidelines of the Institutional Ethics Committee.

C57BL/6 mice were inoculated with 2 × 10^6 ^B16-F10 melanoma cells subcutaneously in the right flank using a disposable tuberculin syringe. The day of inoculation was defined as day 0. Primary palpable tumors developed on day 6-7. On day 8, the tumor bearing mice were randomly assigned into 4 groups and each group contained 8 mice. Two groups received twice daily intraperitoneal (i.p) injections of either PBS or recombinant murine leptin (1 μg/g initial body weight). Two groups received i.p. injections of either 9F8 monoclonal antibody or the control mouse IgG at 50 μg/injection every 3 consecutive days on days 8, 11 after tumor induction. 9F8 is a monoclonal antibody to the human leptin receptor (ObR) which has been developed by Fazeli and Zarkesh-Esfahani and tested for antagonist activity using a leptin signaling bioassay [21]. 9F8 antibody was a kind gift from Professor Richard Ross, Sheffield University, UK. The mouse IgG was kindly gifted by Dr Ali Mostafaei (Medical Biology Research Center, Kermanshah University of Medical Sciences) At the day 14, all animals were euthanized via pentobarbital overdose. Tumors were then carefully dissected, and weighed. Moreover, tumor volumes were calculated as prolate spheroid: V = (4/3*π*(a)2*(b), were "a" is half of the minor axis and "b" is half of the major axis of the prolate spheroid. The weight of the mice was measured immediately after tumor resection.

### Flow cytometry quantification of EPC

Mice were bled through heart puncture for EPC enumeration by flowcytometry. EPC were quantified using the endothelial murine markers VEGF receptor2(PE; R&D Systems,), and CD34(FITC;eBioscience Inc., SanDiego, California)and the CD45 (PerCP;Santa Cruz Biotechnology, Inc., Santa Cruz, California)as described previously with minor changes [22]. Briefly, blood collected in EDTA containing tubes were incubated for 10 minutes with FcR-blocking (miltenyibiotec, Germany). 500 μl of whole blood was incubated with 4 μl of CD45, 8 μl of KDR, and 5 μl of CD34. Respective isotype controls were used as anegativecontrol(eBioscience Inc., SanDiego, California) at 5 μg/ml concentration each. The samples were lysed before flow cytometry analysis.

**Figure 1:**
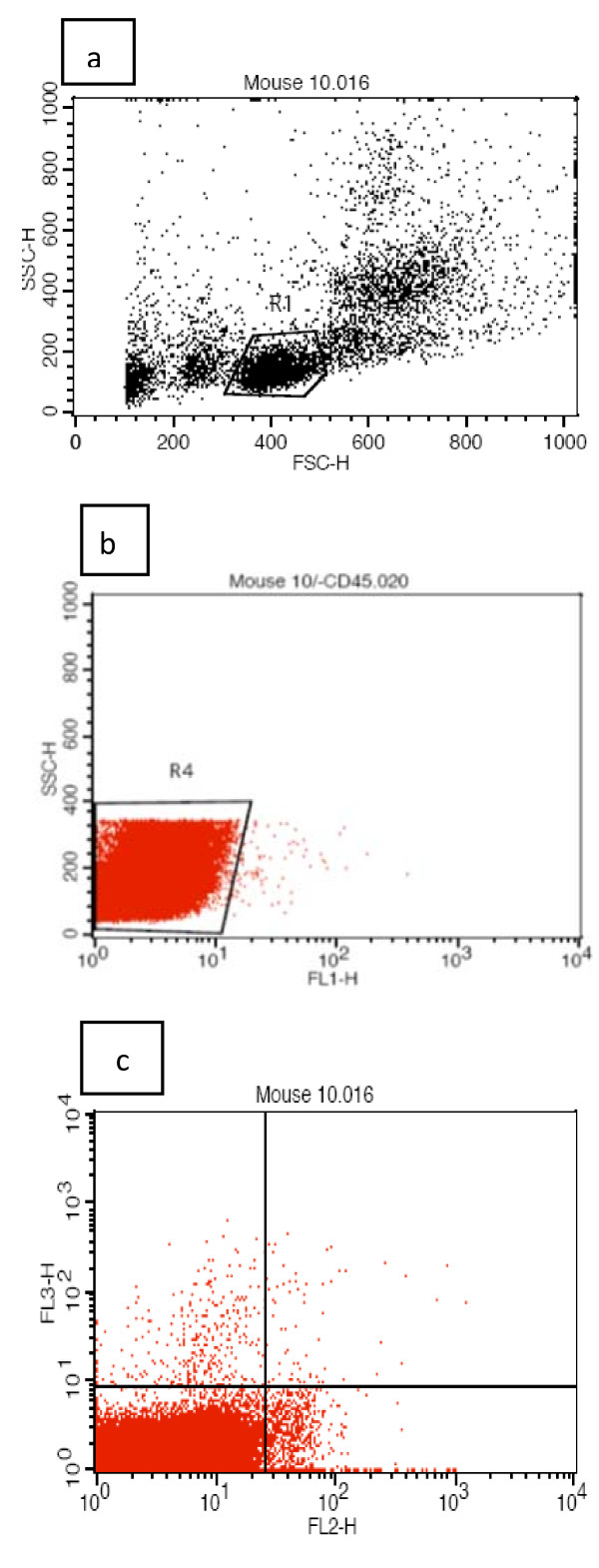
**Characterization of endothelial progenitor cells (EPCs) by flowcytometry evaluation**. First, cells were plotted in forward vs side scatter to gate the lymphocyte population selectively, where EPCs are usually found (a). For analysis of CD45dimCD34+KDR+ endothelial progenitor cells, CD45 was then plotted against the side scatter (b), followed by further analysis of the CD45dim population on coexpression of CD34/KDR (c).

After RBC lysis, cellsuspensions were evaluated by a FACSCalibur (BD Biosciences). The numberof CD45^dim^CD34^+^KDR^+ ^EPCswas determined by a two-dimensional side-scatter fluorescencedot-plot analysis of the sample after gating onthe lymphocyte population (Figure 1). The number of EPCs was expressed per 1 mlblood [22].

### Nitrite and leptin measurement

Mice were fasted for 14 h prior to sacrificing in order to obtain fasted blood samples.

Plasma was isolated from whole blood collected and total nitrite (NOx) was measured (R&D Systems) as an indicator of endothelial release of NO as previously described [23].

Moreover, plasma leptin concentration was measured by ELISA kit (R&D Systems) in mice according to manufacturer's instructions.

### Statistical analysis

Data are expressed as mean ± SD and were tested for normal distribution with the Kolmogorov-Smirnov test. Comparisons between groups were analysed by ANOVA followed by the Bonferroni method as post hoc-test. Differences in the weight of the mice were analyzed using the paired-sample t test. Statistical significance was assumed, if a null hypothesis could be rejected at p ≤ 0.05. All statistical analysis was performed with SPSS 16 (SPSS Inc.).

## Results

**Figure 2:**
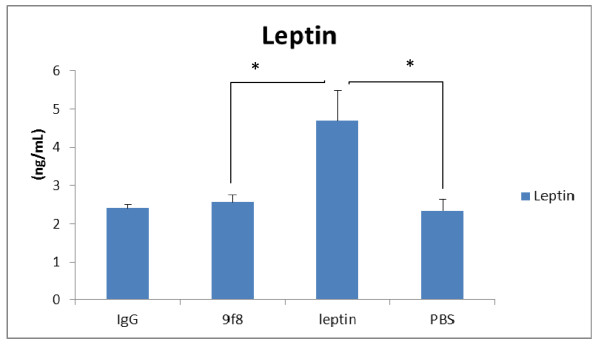
**The plasma levels of leptin were significantly higher in leptin group compared to all other groups of mice while there was no significant difference between other groups**. * (p < 0.05).

The plasma levels of leptin were significantly higher in leptin group compared to all other groups of mice while there was no significant difference between other groups (Figure 2).

Body weights for each group of mice are shown in Table 1. There was a significant weight loss in mice of leptin group while the weight of the animals of 9F8 group increased significantly during the study. By the end of the experiment there was a significant difference between leptin and 9f8 group in body weight and also between each group and its relevant control group.

**Table 1:** The weight of mice in each group of the study.

group	Mice weight1	Mice weight2	P(before-after)
IgG	23.41 ± 0.31	23.24 ± 0.479	p > 0.05

9f8	22.74 ± 0.30	25.37 ± 0.77*	P < 0.05

leptin	22.68 ± 0.99	19.25 ± 1.53*γ	P < 0.05

PBS	24.37 ± 1.22	24.60 ± 1.20	p > 0.05

*Significant difference with respective control group

γ Significant difference with 9F8 group

**Figure 3:**
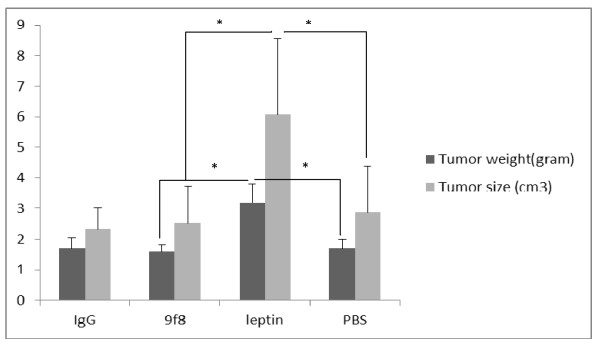
**Mean tumors size and weight**. The weights and volume of melanoma tumors excised from leptin treated mice were significantly larger than tumors from other groups of mice. There was no significant difference between three other study groups. * (p < 0.05).

The melanoma tumor weight of leptin treated mice were significantly more than tumors from other groups of mice while there was no significant difference between other groups (Figure 3).

**Figure 4:**
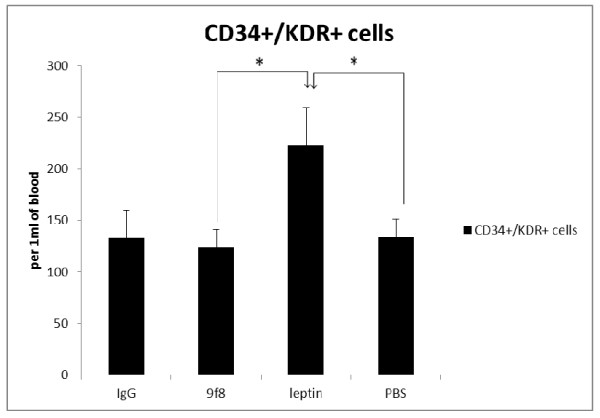
**The circulating EPC numbers**. Leptin treated melanoma tumor bearing mice have more EPCs in peripheral blood than all other study groups. There was no significant difference between three other study groups. * (p < 0.05).

Leptin treatment also resulted in significant more circulating EPCs in tumor bearing mice whereas there was no significant difference between other groups (Figure 4).

**Figure 5:**
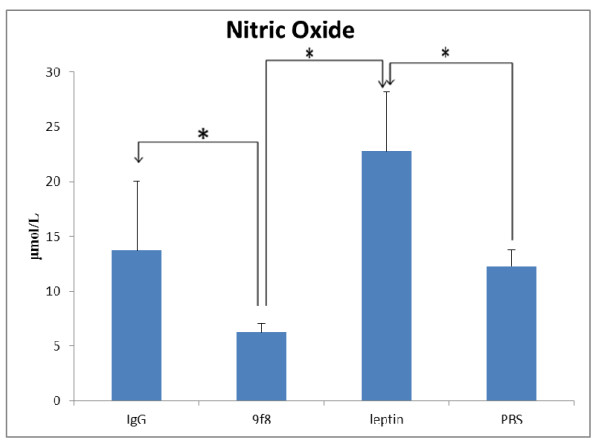
**The plasma concentration of NOx**. The plasma concentration of NOx significantly increased in leptin group and significantly decreased in 9f8 treated mice compare to respective control groups. Furthermore leptin treated mice had significantly more NOx levels than 9F8 group. * (p < 0.05).

The plasma concentration of NOx significantly increased in leptin group and significantly decreased in 9f8 treated mice compare to respective control groups (Figure 5).

## Discussion

Adipose tissue secretes several adipokines that are supposed to stimulate inflammation, cell proliferation and angiogenesis. One of the most important member of such adipokines family is leptin, which increases cell proliferation in several tumor cell lines, enhances endothelial cell migration in vitro, and has been suggested to be an angiogenic/vasculogenic factor [12-17,20].

It has been suggested that leptin may contribute to tumor growth. However, a direct cause and effect role of leptin in accelerating tumor growth is uncertain. Besides, most of the data supporting leptin's role in stimulating cell proliferation and angiogenesis have been derived from *invitro *studies.

In our study, the tumors weight of leptin treated mice were significantly more than tumors from all other groups of mice. Leptin has been identified in several types of human cancers and may also be linked to poor prognosis. In two studies, leptin and leptin receptor expression were significantly increased in primary and metastatic breast cancer relative to noncancerous tissues in women [24]. In a clinical study of colorectal cancer, leptin expression was associated with tumor G_2 _grade [25]. In renal cell carcinomas leptin and leptin receptor expression was well correlated with progression-free survival, venous invasion and lymph node metastasis [26]. Leptin has also been suggested to have a role in uterine and endometrial cancers [27]. There is very little previous information on the relationship between leptin and melanoma. Just one epidemiological study demonstrated that high serum leptin was positively correlated with melanoma risk [19].

The limited published animal studies trying to find whether leptin promote tumor growth have reported different results. Some studies support the hypothesis that the absence of leptin signaling diminishes mammary tumor growth in mice [10,20,28,29].

Brandon et al, in their well-designed study have shown that leptin deficiency attenuates but does not abolish melanoma tumor growth [20].

Furthermore, In mouse model of mammary tumor, using a leptin receptor antagonist [28]revealed that leptin signaling promotes the growth of some types of mammary tumors and increases the expression of proliferating cell nuclear antigen, cyclin D1, vascular endothelial growth factor (VEGF) and its receptor type two (VEGF-R2) [30,31]. Furthermore Fusco et al have recently shown that inactivation of LepR inhibits proliferation and viability of human breast cancer cell lines [32]. Inconsistent with the results of these studies, obese Zucker rats, which have defective leptin receptor, developed more mammary tumors than lean Zucker rats after exposure to the carcinogen, 7,12-dimethylbenzanthracene [33].

Leptin administration led to increase plasma NO concentrations as have been reported previously in several other studies [34-37]. It has been shown that the leptin-induced NO production is mediated through protein kinase A and mitogen-activated protein kinase (MAPK) activation. Interestingly antagonism of leptin by 9f8 antibody resulted in significantly lower plasma NO concentrations compare to both leptin and control group. The significant effect of this antibody on NO production despite of non-significant effects on tumor growth and EPC numbers may be because of use of large, pharmacological concentrations of leptin to demonstrate the 2 latter effects in this study.

Leptin receptors are expressed in mouse melanoma cells as well as EPCs [38].

The results of the present study indicated that leptin enhance the numbers of EPCs in peripheral blood. Recent studies indicated that the EPC derived from bone marrow also contributes to tumor vasculogenesis [3-5,39]. However the extent of EPCs incorporation into the tumor vasculature has been a subject of controversy [40-42]. To the best of our knowledge, this is the first time that has been shown that leptin increased EPCs in melanoma tumor model. It has been recently reported that leptin increased the adhesion and the homing potential of EPCs and may thus enhance their capacity to promote vascular regeneration in vivo [38]. Leptin induces NO, an important mediator of EPC mobilization. NO may trigger EPC recruitment from bone marrow probably by activating a phosphatidylinositol (PI) 3-kinase-independentAkt-eNOS phosphorylation pathway [42,43]. So, the mechanism of increased EPCs in the circulation may be due to mobilization of these cells from bone marrow. Furthermore it has been shown that leptin can increase other mediators of vasculogenesis such as VEGF, and intracellular signaling pathways of cell proliferation, including p38 MAPK and ERK1/2 MAPK phosphorylation [44].

## Conclusion

In conclusion, our observations indicate that leptin causes melanoma growth. The mechanisms by which leptin promotes melanoma growth likely involve increased NO production and circulating EPC numbers and consequently vasculogenesis.

## Competing interests

The authors declare that they have no competing interests.

## Authors' contributions

SHJ had substantial contributions to conception and design, analysis and interpretation of data, and writing the manuscript. FA carried out the cell culture, animal experiment and all other laboratory experiments. HZ and MK had contributions to conception and design. HZ has also been involved in analysis and interpretation of flowcytometry data and drafting the manuscript. MN carried out the flowcytometry measurements. All authors read and approved the final manuscript.
